# Evaluating the potential of graphene oxide to promote skeletal muscle complex regeneration

**DOI:** 10.3389/fbioe.2025.1574145

**Published:** 2025-07-31

**Authors:** Yulu Chen, Zeyu Zhu, Yian Shen, Xuling Liu, Yushi He, Chengqi Lyu, Jiayu Lu

**Affiliations:** ^1^ Department of Stomatology, Shanghai Sixth People’s Hospital Affiliated to Shanghai Jiao Tong University School of Medicine, Shanghai, China; ^2^ Shanghai Electrochemical Energy Devices Research Center, School of Chemistry and Chemical Engineering, Shanghai Jiao Tong University, Shanghai, China

**Keywords:** graphene, exosome, myogenic differentiation, musculoskeletal repair, C2C12

## Abstract

**Background:**

Repair and regeneration of the musculoskeletal system are critical for maintaining mobility, physical function, and overall quality of life. This study aimed to optimize the size and concentration of graphene oxide (GO) to achieve a balance that enhances the proliferation and myogenic differentiation of C2C12 cells and investigate the underlying mechanisms, including the activation of key myogenic genes and signaling pathways. Additionally, the effects of exosomes derived from GO-treated C2C12 myoblasts on osteoblasts were explored.

**Methods:**

C2C12 cells were cultured with different concentrations (0.1, 0.5, 2.5, 12.5, and 62.5 μg/mL) and particle sizes (>500 and <500 nm) of GO. Thereafter, cell viability, proliferation, cycle, and migration were evaluated via fluorescence staining, CCK-8, flow cytometry, and scratch assays, respectively. Immunofluorescence, polymerase chain reaction, and RNA sequencing (RNA-seq) were used to detect the effects of GO on C2C12 cell differentiation and explore the related molecular mechanisms. Furthermore, RNA-seq analysis was performed to investigate the impact of exosomes derived from GO-treated C2C12 myoblasts on MC3T3-E1 cells.

**Results:**

GO with particle sizes of >500 nm at a concentration of 2.5 μg/mL significantly enhanced C2C12 cell proliferation and myogenic differentiation. Increased GO conductivity played a crucial role in supporting *MyoD* expression and promoting myocyte differentiation, likely by modulating membrane electrical activity and facilitating intercellular signaling. These effects were associated with the activation of the PI3K-Akt signaling pathway and the upregulation of the *NFATc1* gene, further highlighting the role of GO’s conductive properties in regulating myogenic differentiation. Exosomes derived from GO-treated myoblasts upregulated genes such as *PDGFRB*, *COL12A1*, and *TBX2* while downregulating inflammation-related genes such as *C3*, thereby demonstrating the crosstalk between muscle and bone cells.

**Conclusion:**

The conductive properties and surface roughness of GO significantly enhanced interactions between muscle and bone tissues, consequently facilitating effective musculoskeletal repair. This study suggests that GO can serve as a promising material for integrated approaches in musculoskeletal tissue engineering by promoting both myogenic differentiation and osteoblastic activity. Our findings highlight the potential utility of GO in regenerative medicine, offering a novel strategy for musculoskeletal regeneration.

## 1 Introduction

Skeletal muscles, which account for 40%–50% of adult body weight, are essential for movement, posture, breathing, and heat production (Laumonier and Menetrey, 2016). Muscle damage caused by trauma, overuse, or degenerative conditions such as Duchenne muscular dystrophy, facioscapulohumeral muscular dystrophy (FSHD), and myotonic dystrophy type 1 (DM1) can lead to severe pain, loss of mobility, and a markedly reduced quality of life (Duan et al., 2021; Papaefthymiou et al., 2022; Dumont et al., 2015). Over the past 3 decades, musculoskeletal disorders have increased by 30% (Global, 2019; Safiri et al., 2021). Muscle repair relies on satellite cells, which are activated by signals from the damaged environment to migrate to the injury site, proliferate, and regenerate muscle tissue (Liu et al., 2018). However, this regenerative capacity is limited, allowing compensation for up to 20% of muscle mass loss; beyond this point, its adaptive and regenerative potential becomes inadequate (Liu et al., 2018; Corona et al., 2015). Repair is further complicated by challenges such as incomplete regeneration, scar tissue formation, chronic inflammation, and limited vascularization, hindering effective healing. This regenerative ability is impaired in conditions such as muscular dystrophy, leading to muscle dysfunction. Furthermore, aging and poor integration with surrounding tissues impede recovery, highlighting the urgent need for novel therapies that enhance muscle regeneration and address these challenges to effectively restore muscle function (Moiseeva et al., 2023).

The musculoskeletal system comprises bones, muscles, tendons, ligaments, and other connective tissues that work together to support the body structure, enable movement, and maintain stability. Bone and muscle tissues interact in a dynamic manner, with muscle contraction exerting forces on bones through tendons, allowing joint movement. This interaction is essential for mobility, posture, and overall function. Furthermore, skeletal muscles and bones are influenced by shared biochemical and biomechanical signals that regulate their development, maintenance, and response to injury (Herrmann et al., 2020). Disruption in the interaction between muscles and bones, often observed in conditions such as osteoporosis, muscular dystrophies, and age-related degeneration, can impair the ability of the musculoskeletal system to maintain function and stability (He et al., 2020; Catalano et al., 2022; Chagarlamudi et al., 2017; Gielen et al., 2023; Lu et al., 2018).

Current repair strategies for the musculoskeletal system primarily focus on addressing bone and muscle injuries independently. For bone repair, these strategies include the use of bone grafts, synthetic scaffolds, and osteogenic growth factors to promote healing (Safari et al., 2021; Stamnitz and Klimczak, 2021). Meanwhile, muscle repair is typically approached through the activation of satellite cells, myoblast transplantation, and the use of biomaterials to stimulate myogenic differentiation (Sousa-Victor et al., 2022; Nuge et al., 2021). Although these approaches have shown some success, they often fail to address the complex interconnected nature of muscle–bone interactions. For example, bone repair strategies often overlook the role of muscle strength and function in maintaining bone health, whereas muscle repair techniques do not sufficiently account for the influence of bones on muscle regeneration. Furthermore, numerous existing therapies do not fully restore function, particularly in degenerative conditions such as muscular dystrophies or age-related sarcopenia, wherein both muscles and bones are compromised. The complexity of these interactions presents challenges in developing therapies that target both systems simultaneously, and more research is necessary to optimize such integrated approaches.

Graphene oxide (GO) exhibits exceptional physical properties, including higher hardness than diamond, an elastic modulus of up to 1 TPa, and a tensile strength of ∼130 MPa (Lee et al., 2008). These properties render GO ideal for simulating the mechanical strength and elasticity of skeletal muscles. Furthermore, its nanoscale roughness effectively mimics that of natural extracellular matrix (ECM), which can promote protein adsorption and cell adhesion (Bacakova et al., 2011). The π–π interactions and the presence of oxygen-containing functional groups on GO enhance its ability to bind proteins, further facilitating muscle cell adhesion and differentiation (Kenry et al., 2018; Luong-Van et al., 2020). Skeletal muscles are highly responsive to electrical signals, and processes such as cell communication, proliferation, differentiation, and contraction depend on these stimuli. The excellent electrical conductivity of GO, resulting from its sp^2^ hybridized carbon structure and free-moving π electrons, can influence gene expression, thereby enhancing muscle cell adhesion, proliferation, and differentiation (Jung et al., 2008; Dong et al., 2020; Han et al., 2023; Chen et al., 2013).

In addition to its effects on muscle cells, GO has demonstrated considerable potential in bone regeneration. International studies have reported that GO enhances osteoblast proliferation and differentiation, thereby improving bone mineralization and accelerating bone repair processes (Bahrami et al., 2021; Berrio et al., 2021; Wong et al., 2020; Ș et al., 2022). MC3T3-E1 cells are osteoblastic precursor cells derived from mouse calvaria that can differentiate into mature osteoblasts to form a mineralized bone matrix under appropriate conditions. These cells are extensively used in bone biology research because of their relevance in osteogenesis and bone tissue repair (Izumiya et al., 2021; Seo et al., 2010; Fukuda et al., 2013).

To investigate the impact of GO on skeletal muscle proliferation and differentiation and the effects of the interplay between muscles and bones (musculoskeletal crosstalk), this study evaluated the effects of GO on C2C12 cells, examining how different GO concentrations and sizes influence cell viability, migration, and differentiation. GO was found to promote the intracellular flow of calcium ions through its electrical conductivity, in turn activating the NFATc1 signaling pathway, which leads to the upregulation of MyoD and promotes myogenic differentiation. Furthermore, we explored the effects of exosomes derived from GO-treated C2C12 cells on MC3T3-E1 cells, highlighting GO’s potential for comprehensive musculoskeletal regeneration. This dual focus emphasizes the interconnectedness of muscle and bone regeneration and highlights potential pathways through which GO can promote comprehensive musculoskeletal repair, thereby offering a holistic approach to addressing musculoskeletal disorders.

## 2 Methods

### 2.1 Characterization and conductivity

The GO used in this research was purchased from Nano Xianfeng Company, China. We observed the shape and size of GO using scanning electron microscopy (SEM) (Tescan MIRA, Czech Republic) and measured its surface roughness using atomic force microscopy (AFM) (PARK NX-Hivac, Korea).

The samples with particle sizes greater and less than 500 nm were diluted to concentrations of 0.1, 0.5, 2.5, 12.5, and 62.5 μg/mL, respectively, following a concentration gradient. The conductivity of each dilution was subsequently measured using a conductivity detector (HANNA HI8733, Italy).

### 2.2 Cell culture

The C2C12 cell line was purchased from Procell (CL-0044, China) and cultured in Dulbecco’s Modified Eagle Medium (DMEM) (HyClone, United States) supplemented with 10% fetal bovine serum (HyClone) and 1% penicillin–streptomycin (HyClone). The cells were maintained at 37°C in a humidified incubator containing 5% CO_2_ (Zhang et al., 2023). The medium was replaced every 2–3 days to ensure optimal growth conditions. The cells were passaged when they reached approximately 80%–90% confluence. All subsequent cell culture experiments were performed with at least three biological replicates (*n* = 3) to ensure reproducibility.

### 2.3 Cell viability assay

To evaluate the effects of different concentrations and sizes of GO on the viability of C2C12 cells, these cells were seeded in 96-well plates (NEST, United States) at a density of 1 × 10^4^ cells per well. The cells were allowed to adhere for 12 h and then treated with two types of GO differing in sheet size: one with diameters of >500 nm and the other with diameters of <500 nm. The treatments were administered at concentrations of 0, 0.1, 0.5, 2.5, 12.5, and 62.5 μg/mL. On days 1, 3, and 5 after treatment, 10 µL of the CCK-8 reagent (Beyotime Biotechnology, China) was added to each well containing cells in a 96-well plate. The cells were then incubated with the reagent for 2 h. The absorbance measured by the microplate reader was recorded for further analysis to assess the effects of both types of GO on cells over time.

The C2C12 cells were seeded into 24-well plates (NEST) at a density of 2 × 10^4^ cells per well and allowed to adhere. Following cell attachment, the cells were treated with two types of GO (diameters >500 nm and diameters <500 nm) at concentrations of 0, 0.1, 0.5, 2.5, 12.5, and 62.5 μg/mL with particle sizes of either >500 nm or <500 nm. The cells were incubated for 24 h, following which they were washed with phosphate-buffered saline (PBS). Subsequently, the cells were stained using a live/dead cell staining kit (ScienCell, United States) and photographed with a fluorescence microscope (Olympus CKX53, Japan).

### 2.4 Flow cytometric analysis

The C2C12 cells were seeded in six-well plates at a density of 1 × 10^5^ cells per well. They were treated with different concentrations of GO, particularly 0.1, 0.5, 2.5, and 62.5 μg/mL, with particle sizes of either >500 nm or <500 nm. Following a 24-h incubation period, the cells were harvested, fixed in 70% ethanol, and stained with propidium iodide (PI) to label DNA. Flow cytometric analysis was performed to determine the percentage of cells in each phase of the cell cycle—G0/G1, S, and G2/M.

The C2C12 cells were seeded into six-well plates at a density of 1 × 10^5^ cells per well. Upon reaching 70%–80% confluence, the cells were treated with varying concentrations of GO (0.5 and 2.5 μg/mL) and particle sizes of >500 and <500 nm. After 48 h of treatment, the cells were washed thrice with Hank’s balanced salt solution (HBSS, Siwega) and then incubated with Fluo-4 AM working solution (YEASEN, China). After incubation, the cells were washed thrice with HBSS and then further incubated in HBSS at 37°C for an additional 30 min to allow the complete uptake of Fluo-4 AM. Subsequently, the adherent cells from each treatment group were trypsinized, suspended in a medium, and centrifuged at 1,000 rpm for 5 min. The supernatant was discarded, and the cells were resuspended in PBS. The suspension was centrifuged again at 1,000 rpm for 5 min. This washing step was repeated 1 to 2 times. Finally, the cells were transferred to flow cytometry tubes for detection.

### 2.5 Cell migration

The C2C12 cells were cultured in six-well plates until they reached ∼90% confluence. A sterile 200-µL pipette tip was then used to create a straight scratch in each well to simulate a wound. The cells were gently washed with PBS to remove detached cells and debris, and fresh culture medium was added, followed by treatment with two types of GO with different sheet sizes (diameters >500 nm and diameters <500 nm) at concentrations of 0.5 and 2.5. Images were captured using a Nikon DS-Qi2 optical microscope immediately after scratching and 24 h after scratching. To assess the scratch width at 0 and 24 h, six measurement points in the image were selected using ImageJ software. The cell migration rate was then calculated using the following formula: migration rate (%) = [(Scratch width at 0 h − Scratch width at 24 h)/Scratch width at 0 h] × 100%.

### 2.6 F-actin staining

The C2C12 cells were seeded in a 12-well plate (NEST, United States) with round coverslips (Biosharp, China) at a density of 1 × 10^5^ cells per well. Subsequently, the cells were treated with two types of GO with different sheet sizes at concentrations of 0.5 and 2.5 μg/mL until they reached 70% confluence. The cells were fixed with 4% paraformaldehyde in PBS for 10 min at room temperature (24°C). Then, the cells were permeabilized with 0.1% Triton X-100 (BioFroxx, Germany) in PBS for 5 min. After permeabilization, the cells were incubated with a blocking solution of 3% bovine serum albumin (BSA) (Beyotime, China) in PBS for 30 min to prevent nonspecific binding. Actin-Tracker Red (Beyotime, China) was then diluted with secondary antibody diluent at a ratio of 1:150, and 200 µL of the solution was added to each sample. The mixture was then incubated for 60 min at room temperature in the dark. The cells were subsequently washed with PBS and counterstained with 4ʹ,6-diamidino-2-phenylindole (DAPI) to visualize the nuclei. Images were captured using a fluorescence microscope (Zeiss Axio Imager A2, Germany). From the obtained fluorescence images, cell width, length, and aspect ratio data were analyzed using ImageJ. The aspect ratio was calculated as follows: Aspect Ratio = Cell Length/Cell Width.

### 2.7 Cell differentiation

The cell viability assay results indicated that GO at concentrations of ≤2.5 μg/mL exhibited good biocompatibility. The C2C12 cells were differentiated in DMEM containing 2% horse serum (Procell, China) and 1% penicillin–streptomycin. The experimental groups were treated with GO having diameters of >500 nm and <500 nm at concentrations of 0.1, 0.5, and 2.5 μg/mL, with a control group lacking GO. Medium changes were performed every 2–3 days.

#### 2.7.1 Real-time polymerase chain reaction

Based on the biocompatibility assessments results, 5 days after the C2C12 cells were treated with two types of GO with different sheet sizes (diameters >500 nm and diameters <500 nm) at concentrations of 0.5 and 2.5 μg/mL, the cells were collected for real-time polymerase chain reaction (PCR) to assess gene expression associated with muscle differentiation. The cells were collected and lysed using TRIzol reagent, and the total RNA was isolated according to the manufacturer’s protocol. The RNA concentration was measured using a NanoDrop One spectrophotometer (NanoDrop Technologies, United States). Complementary DNA (cDNA) was synthesized from 1 µg of RNA using a reverse transcription kit (Vazyme, China) according to the manufacturer’s instructions. Real-time PCR was subsequently performed using specific primers for the muscle differentiation markers MyoD (Sangon Biotech, China) and NFATc1 (Sangon Biotech), along with a SYBR Green or TaqMan PCR master mix. The PCR reactions were run on a real-time PCR machine under appropriate thermal cycling conditions. The relative gene expression levels were quantified using the 2^−ΔΔ*C*^_T_method, with glyceraldehyde-3-phosphate dehydrogenase used as the internal control. Data were analyzed to compare the effects of different treatment conditions on the expression of muscle-specific genes.

#### 2.7.2 Immunofluorescence assay

According to the PCR results, the C2C12 cells were differentiated and cultured for 5 days at a concentration of 2.5 μg/mL, and MyHC expression was analyzed using immunofluorescence. The experimental groups were as follows: GO (>500 nm, 2.5 μg/mL) with horse serum, GO (<500 nm, 2.5 μg/mL) with horse serum, GO (>500 nm) without horse serum, GO (<500 nm) without horse serum, and a control group without GO. The cells were then treated as described in Section 2.6 for 5 days to promote myotube formation. Following the differentiation period, the cells were fixed with 4% paraformaldehyde at 24°C for 15 min. The cells were permeated with 0.1% Triton X-100 (Bio Froxx, Germany) for 10 min. The cells were blocked with 3% BSA for 30 min to prevent nonspecific binding. The primary antibody against MyHC (1:200, ABclonal, China, myosin heavy chain, a marker of muscle development and differentiation) was incubated with the cells overnight at 4°C. Then, the cells were incubated with a fluorescently labeled secondary antibody (1:500, Jackson, United States) for 1 h at room temperature in the dark. Following additional washes, the nuclei were stained with DAPI for 5 min. Images were captured using a fluorescence microscope (LEICA DM2000LED, China) and analyzed with ImageJ.

### 2.8 MC3T3-E1 cell treatment

The C2C12 cells were cultured in six-well plates in DMEM supplemented with 2% horse serum to induce differentiation. This setup served as the control group. For the experimental group, the cells were cultured under identical conditions but with the addition of GO (diameter >500 nm, 2.5 μg/mL) to assess its impact on exosome production. After 5 days of treatment, the culture media from both the control and experimental groups were collected separately, and the exosomes were isolated using ultracentrifugation. The media were centrifuged at 300 × g for 10 min to remove cell debris, followed by centrifugation at 10,000 × g for 30 min to pellet the exosomes. MC3T3-E1 cells (CL-0710, Pricella) were cultured in the MC3T3-E1 cell culture medium (CM-0710, Pricella) until they reached 70%–80% confluence.

The exosomes derived from the C2C12 cells, which were cultured with or without GO, were added to the MC3T3-E1 cells at a concentration of 50 μg/mL and incubated for 48 h. In the experimental group, the MC3T3-E1 cells were treated with the exosomes derived from the C2C12 cells exposed to GO and 2% horse serum, whereas the control group received exosomes from the C2C12 cells cultured with 2% horse serum alone.

### 2.9 RNA sequencing analysis

Based on the finding that GO at a concentration of 2.5 μg/mL and particle sizes of >500 nm can promote the proliferation and myogenic differentiation of C2C12 cells, this GO size and concentration were used for RNA sequencing (RNA-seq). The C2C12 cells were differentiated under three conditions: (A) 2% horse serum, (B) a combination of 2% horse serum and GO (diameter >500 nm, 2.5 μg/mL), and (C) GO (diameter >500 nm, 2.5 μg/mL). After a 5-day differentiation period, the cells from each condition were collected for RNA-seq analysis.

In a parallel experiment, the MC3T3-E1 cells were exposed to the exosomes derived from the C2C12 cells cultured under the aforementioned conditions. Following 48 h of exosome treatment, the MC3T3-E1 cells were harvested, and the total RNA was extracted using a commercial RNA extraction kit according to the manufacturer’s instructions. RNA quality and quantity were assessed using a NanoDrop spectrophotometer and an Agilent Bioanalyzer. The RNA samples were subsequently prepared for sequencing by generating libraries and performing RNA-seq using Illumina. The resulting raw sequencing data were analyzed to determine gene expression levels.

### 2.10 Statistical analysis

All data are expressed as means ± standard deviations. The statistically significant differences between groups were analyzed using one-way or two-way analysis of variance in GraphPad Prism 9.5. Differences with *p-*values of <0.05 were considered statistically significant.

## 3 Results

### 3.1 Characterization of different sheet sizes of GO

**FIGURE 1:**
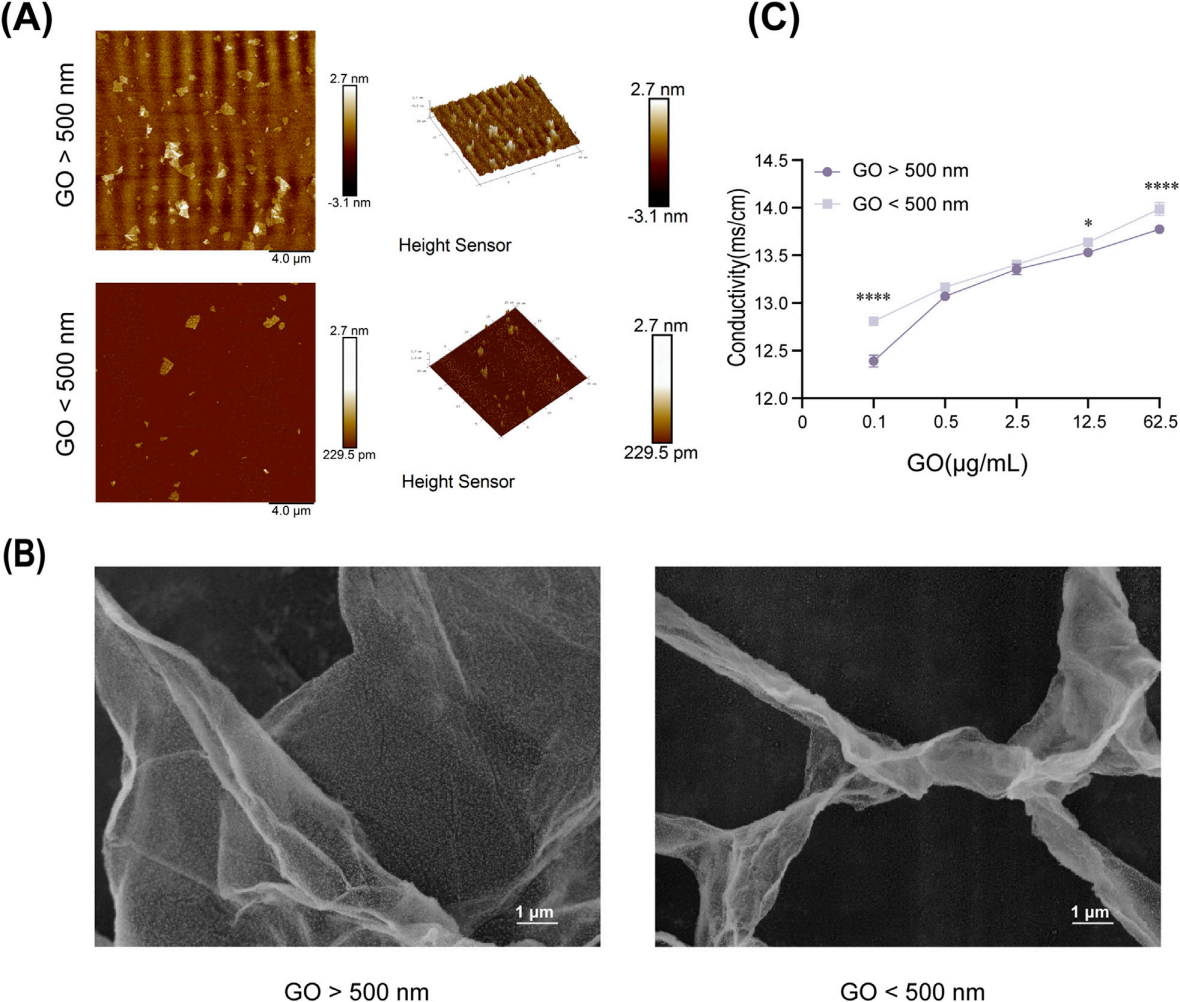
Physical and chemical properties of graphene oxide (GO). **(A)** Atomic force microscopy images of GO (diameter >500 nm) and GO (diameter <500 nm). Scale bars = 4 μm. **(B)** Scanning electron microscopy images of GO at ×10,000 magnification. **(C)** Conductivity of GO at different concentrations (0.1–62.5 μg/mL) and diameters (*P < 0.05, ****P < 0.0001).

The AFM and SEM results revealed clear differences in the surface morphology and structure of GO sheets of various sizes. The larger GO sheets exhibited a more uniform surface topology, whereas the smaller GO sheets displayed more irregular structures (Figures 1A,B).

In terms of conductivity, measurements indicated that the electrical conductivity of GO significantly improved with increasing concentration, regardless of the sheet size (Figure 1C). At the same concentration, smaller GO sheets exhibited slightly higher electrical conductivity than larger GO sheets, possibly because of their increased surface area and better charge mobility (Kim et al., 2017).

### 3.2 Effects of GO on C2C12 cell viability

**FIGURE 2:**
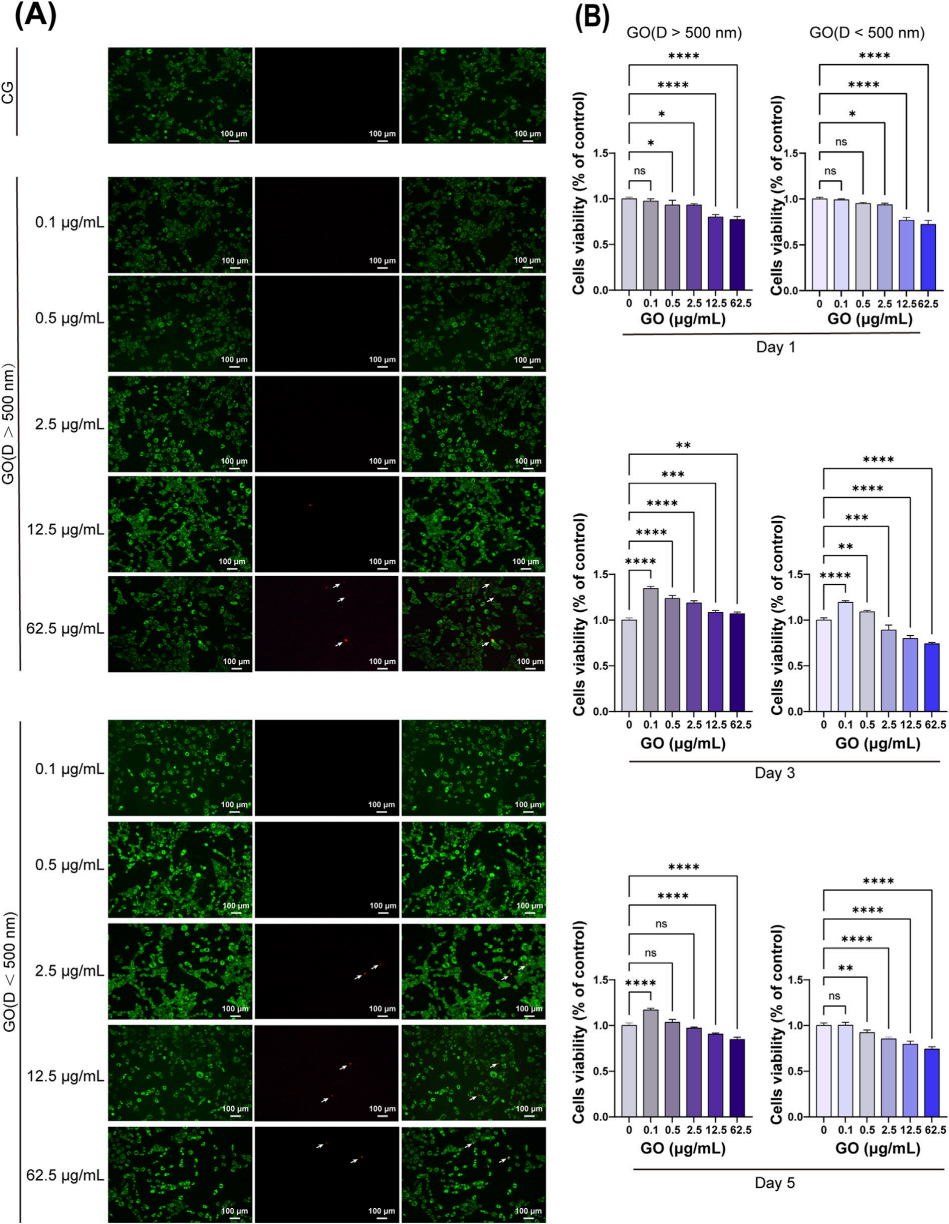
Cytotoxicity of graphene oxide (GO) with different concentrations and diameters. **(A)** Fluorescence images show the results of the live/dead staining with viable cells appearing green and dead cells (white arrow) appearing red. Scale bars = 100 μm. CG represents the conventional culture medium control group without GO. **(B)** CCK-8 assay results on days 1, 3, and 5 (*n* = 4, *p < 0.05, **p < 0.01, ***p < 0.001, ****p < 0.0001).

In the CCK-8 assay evaluating the effects of GO on C2C12 cells, on the third day of culture, low concentrations of GO with sheet diameters of >500 nm were found to promote C2C12 cell proliferation (Figure 2B). By the fifth day, GO at concentrations of ≤2.5 μg/mL did not exert cytotoxic effects, indicating good biocompatibility at lower doses. However, at concentrations of ≥12.5 μg/mL, GO induced significant cytotoxicity, reducing cell viability. Moreover, smaller GO sheets (<500 nm) were found to be more cytotoxic than larger sheets, likely because of their higher surface area and increased interaction with cellular membranes. These results highlight the importance of the size and concentration of GO in determining its biocompatibility with C2C12 cells. Low concentrations of larger GO sheets appeared to support cell growth, whereas higher concentrations and smaller sheets exerted cytotoxic effects.

Live and dead staining experiments were conducted on myoblasts cultured for 1 day with various concentrations and sizes of GO. The results demonstrated that GO concentrations from 0.1 μg/mL to 12.5 μg/mL with particle sizes of >500 nm did not cause cell death, similar to that observed in the control group (Figure 2A). However, at a concentration of 62.5 μg/mL, few dead cells were detected. For GO with particles sizes of <500 nm, concentrations ranging from 0.1 μg/mL to 2.5 μg/mL did not induce cell death; however, at concentrations of >12.5 μg/mL, costaining with both calcein-AM and PI was observed. This costaining indicates that some cells exhibited compromised membrane integrity while still being capable of metabolizing calcein-AM, suggesting that these cells are in a state of stress or early apoptosis.

**FIGURE 3:**
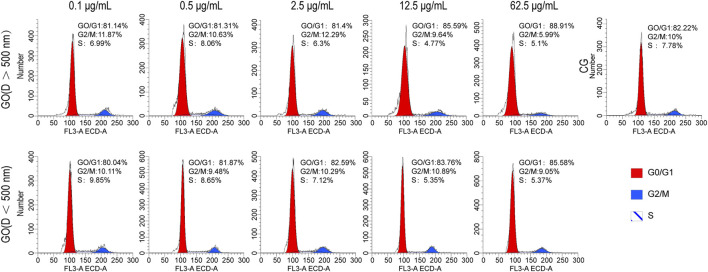
Cell cycle analysis of C2C12 cells treated with graphene oxide (GO); CG represents conventional culture medium control group without GO.

The flow cytometry analysis of C2C12 myoblasts cultured with varying concentrations and diameters of GO for 1 day revealed that when the concentration of GO was <12.5 μg/mL, its impact on cell cycle progression was relatively mild compared with that in higher concentrations. At 2.5 μg/mL, the distribution of cells in the G0/G1, G2/M, and S phases closely mirrored that of the control group, indicating minimal interference with the cell cycle (Figure 3). However, at a higher concentration of 62.5 μg/mL, a notable increase in the number of cells in the G0/G1 phase and a corresponding decrease in the number of cells in the G2/M and S phases were observed, suggesting that elevated concentrations of GO induce cell cycle arrest at the G0/G1 phase, potentially limiting cell division.

### 3.3 Effects of GO on C2C12 cell migration

**FIGURE 4:**
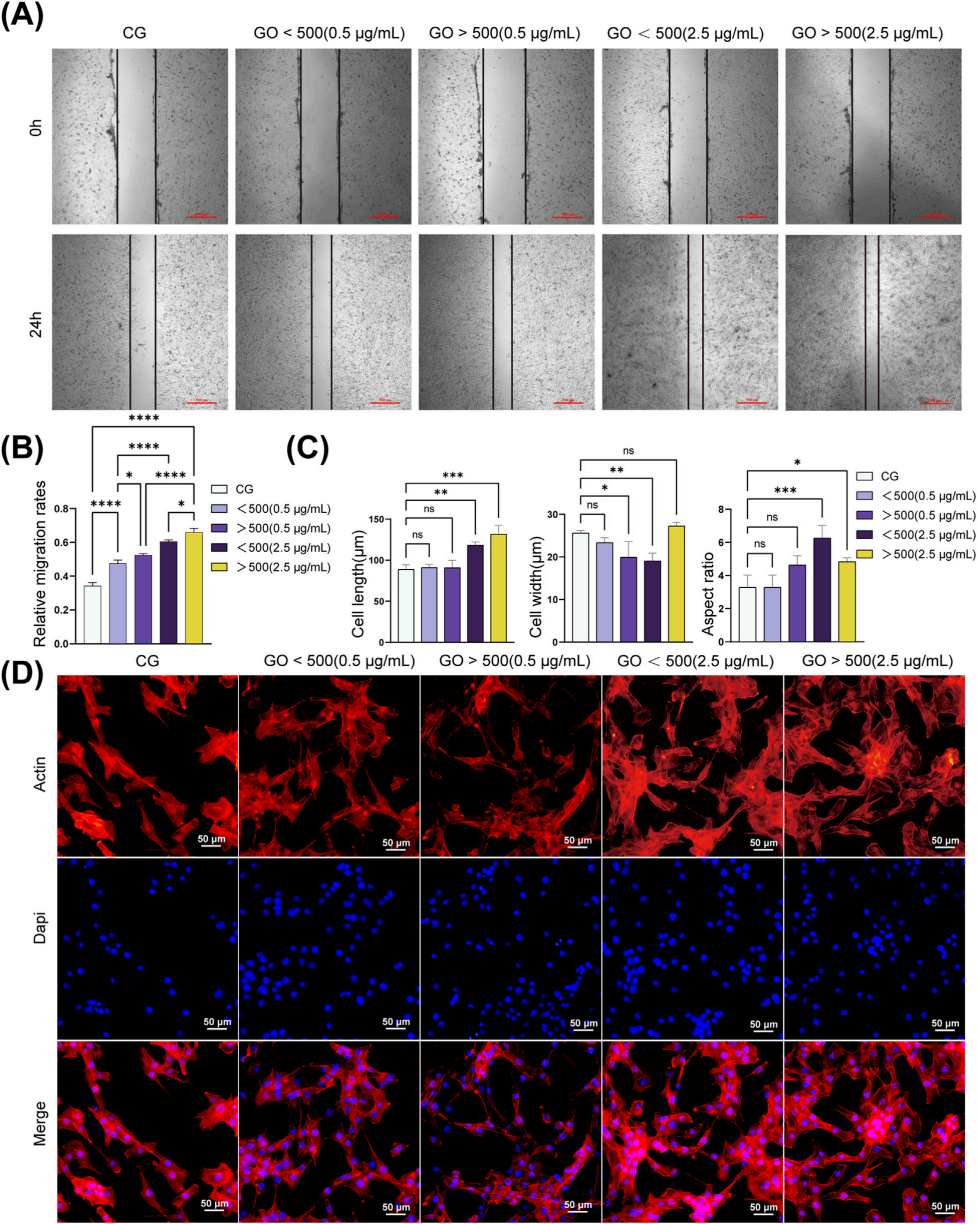
Migration and cytoskeleton of C2C12 cells treated with graphene oxide (GO). **(A,B)** Representative images and quantitative analysis of the scratch assay in C2C12 cells. (**p < 0.01, ***p < 0.001, ****p < 0.0001). **(C)** Morphology analysis of C2C12 cells. (*p < 0.05, **p < 0.01, ***p < 0.001; n = 50 cells per condition). **(D)** Cytoskeleton of C2C12 cells treated with GO. Red: F-actin (phalloidin), blue: nuclei (DAPI). CG represents conventional culture medium control group without GO. Scale bars = 4 μm.

According to the CCK8 results, the control group without GO was compared with the groups treated with GO at 0.5 and 2.5 μg/mL (<500 nm and >500 nm). The cell migration analysis revealed that treatment with GO enhanced the migration rate of myoblasts compared with that in the control group without GO. Alternatively, treatment with 2.5 μg/mL GO with particle sizes of >500 nm resulted in a more pronounced increase in myoblast migration, significantly enhancing cellular motility compared with the that in control group and the 0.5 μg/mL condition (Figures 4A,B). These results suggest that GO influences myoblast migration in a concentration-dependent manner, with higher concentrations of larger GO sheets (diameter >500 nm, 2.5 μg/mL) exerting a more substantial effect on promoting cell mobility.

### 3.4 Effects of GO on the cytoskeleton of C2C12 cells

Cytoskeletal experiments revealed that GO influenced the cytoskeletal organization and morphology of C2C12 cells in a concentration-dependent manner. In the control group without GO, the actin filaments were well-organized, supporting the typical spindle-shaped morphology of myoblasts with moderate migration. At a concentration of 0.5 μg/mL, both smaller GO sheets (diameter <500 nm) and larger GO sheets (diameter >500 nm) slightly enhanced cytoskeletal integrity and motility, with well-maintained actin filaments and improved cell spreading (Figure 4D). At 2.5 μg/mL, the GO sheets, particularly those with diameters of >500 nm, promoted cytoskeletal reorganization, resulting in pronounced stress fiber formation, enhanced cell adhesion, and a substantial improvement in cell migration. Quantitative analysis of the cytoskeleton revealed that treatment with 0.5 μg/mL GO exerted minimal effects on both cell length and width. At a concentration of 2.5 μg/mL, an increase in the cell length and aspect ratio was observed. Notably, GO with diameters of >500 nm increased the cell width, whereas GO with diameters of <500 nm decreased the cell width, accompanied by an increase in the aspect ratio (Figure 4C).

### 3.5 Effects of GO on calcium ion flow in C2C12

**FIGURE 5:**
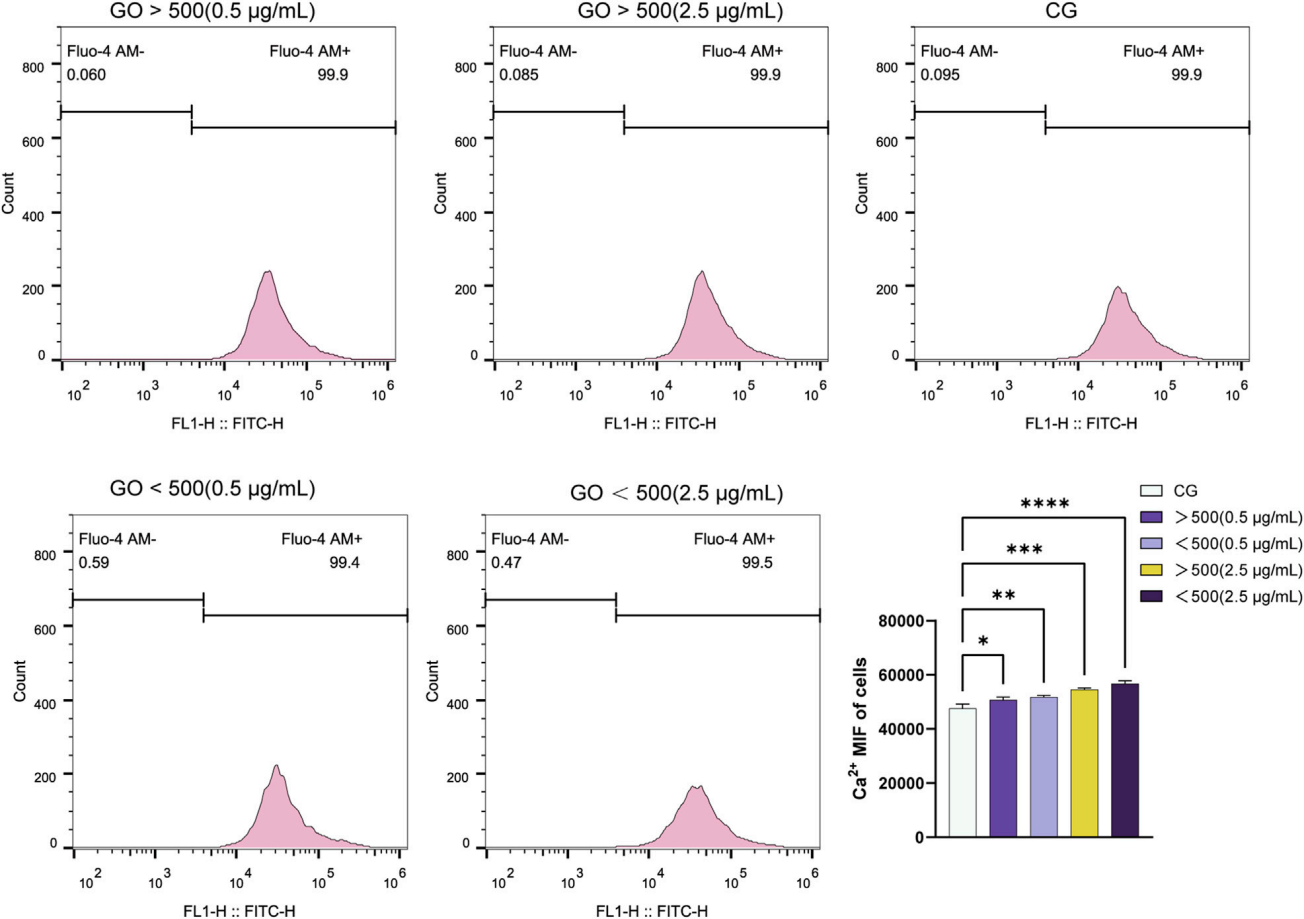
Fluorescence curve and quantitative analysis of calcium ion flux in C2C12 myoblasts treated with graphene oxide (GO) (*p < 0.05, **p < 0.01, ***p < 0.001, ****p < 0.0001).

Flow cytometry analysis of calcium ion flux revealed that higher concentrations of GO, particularly at 2.5 μg/mL, induced greater fluorescence intensity of calcium ions than at concentration of 0.5 μg/mL. Moreover, the particle size of GO influenced the fluorescence intensity, with GO with diameters of <500 nm exhibiting slightly higher calcium ion flux than GO with diameters of >500 nm at the same concentration (Figure 5). Cells treated with 2.5 μg/mL GO—particularly the smaller-sized GO sheets (<500 nm)—showed more pronounced calcium signaling responses. This finding suggests that increased conductivity associated with increased GO concentration and surface area (for smaller particles) enhanced calcium ion influx. The enhanced electrical conductivity of GO appears to facilitate the entry of calcium ions through the cell membrane, likely by modulating ion channel activity, which in turn activates intracellular signaling pathways.

### 3.6 Effects of GO on C2C12 cell differentiation

**FIGURE 6:**
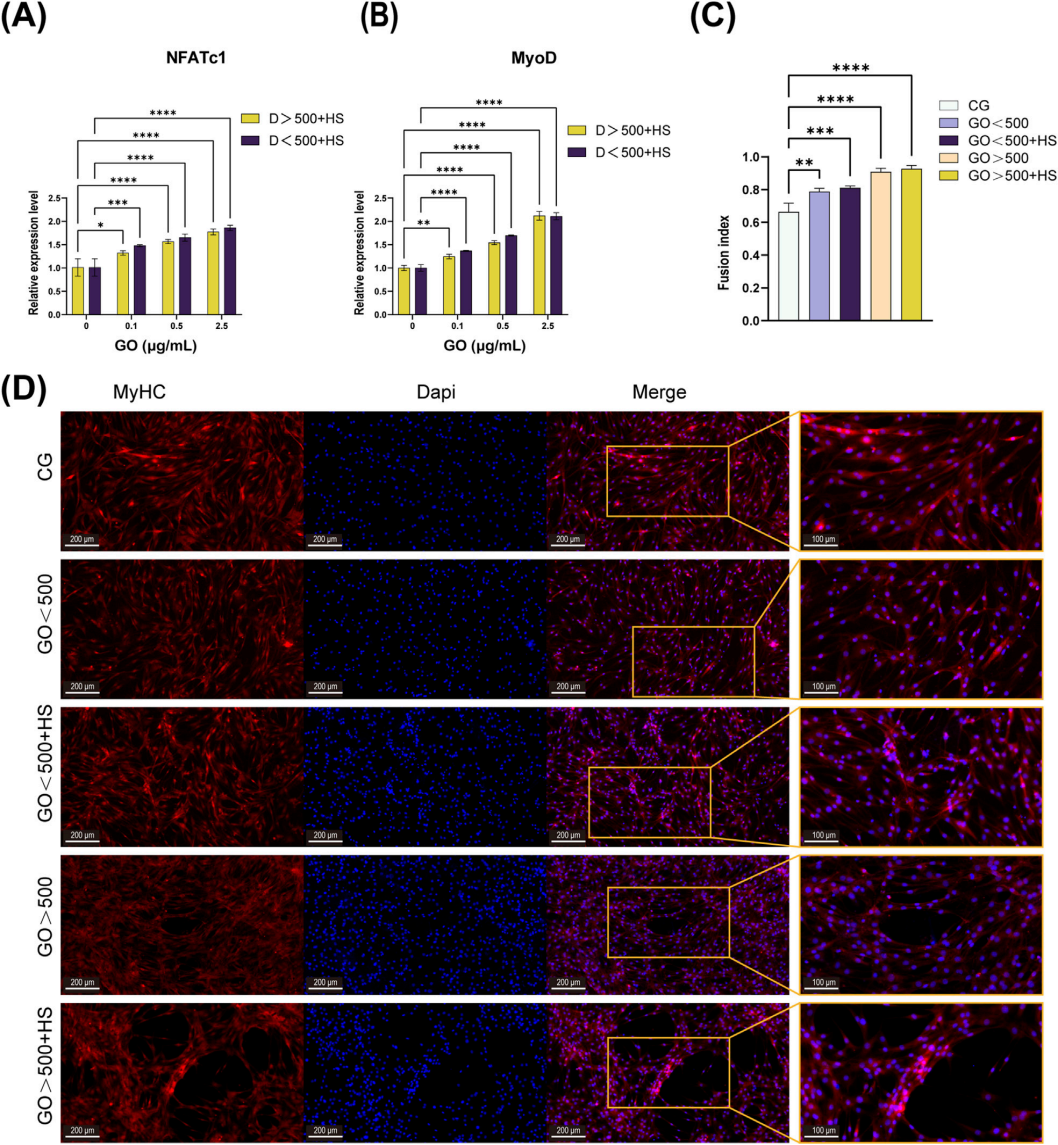
Effects of graphene oxide (GO) on C2C12 cell differentiation. **(A,B)** Polymerase chain reaction (PCR) results of *NFATc1* and *MyoD* after 5 days of differentiation in cultures with only horse serum and GO combined with horse serum (*n* = 3, *p < 0.05, **p < 0.01, ****p < 0.0001). **(C,D)** Immunofluorescence image and fusion index analysis of MyHC after 5 days of differentiation with or without GO (**p < 0.01, ***p < 0.001, ****p < 0.0001). CG means a control group containing only 2% horse serum without GO.

PCR was performed to assess the expression of the *NFATc1* and *MyoD* genes. The results demonstrate a synergistic effect between GO and horse serum, leading to a significant upregulation of *MyoD* expression. At concentrations below 2.5 μg/mL, the expression levels of *NFATc1* and *MyoD* increased in correlation with the electrical conductivity of GO. However, at a concentration of 2.5 μg/mL, GO with a larger size induced higher *MyoD* expression compared to its smaller counterpart (Figures 6A,B).

Immunofluorescence analysis revealed that GO combined with 2% horse serum enhanced the formation of multinucleated myotubes and promoted myoblast fusion, regardless of the GO diameter (Figure 6C). Meanwhile, GO with diameters of >500 nm combined with 2% horse serum not only synergistically promoted the formation of multinucleated myotubes but also increased the length and diameter of the myotubes (Figure 6D). These results suggest that GO size, along with the differentiation medium, influences both the fusion process and the elongation of myotubes, with larger GO having a more pronounced effect.

**FIGURE 7:**
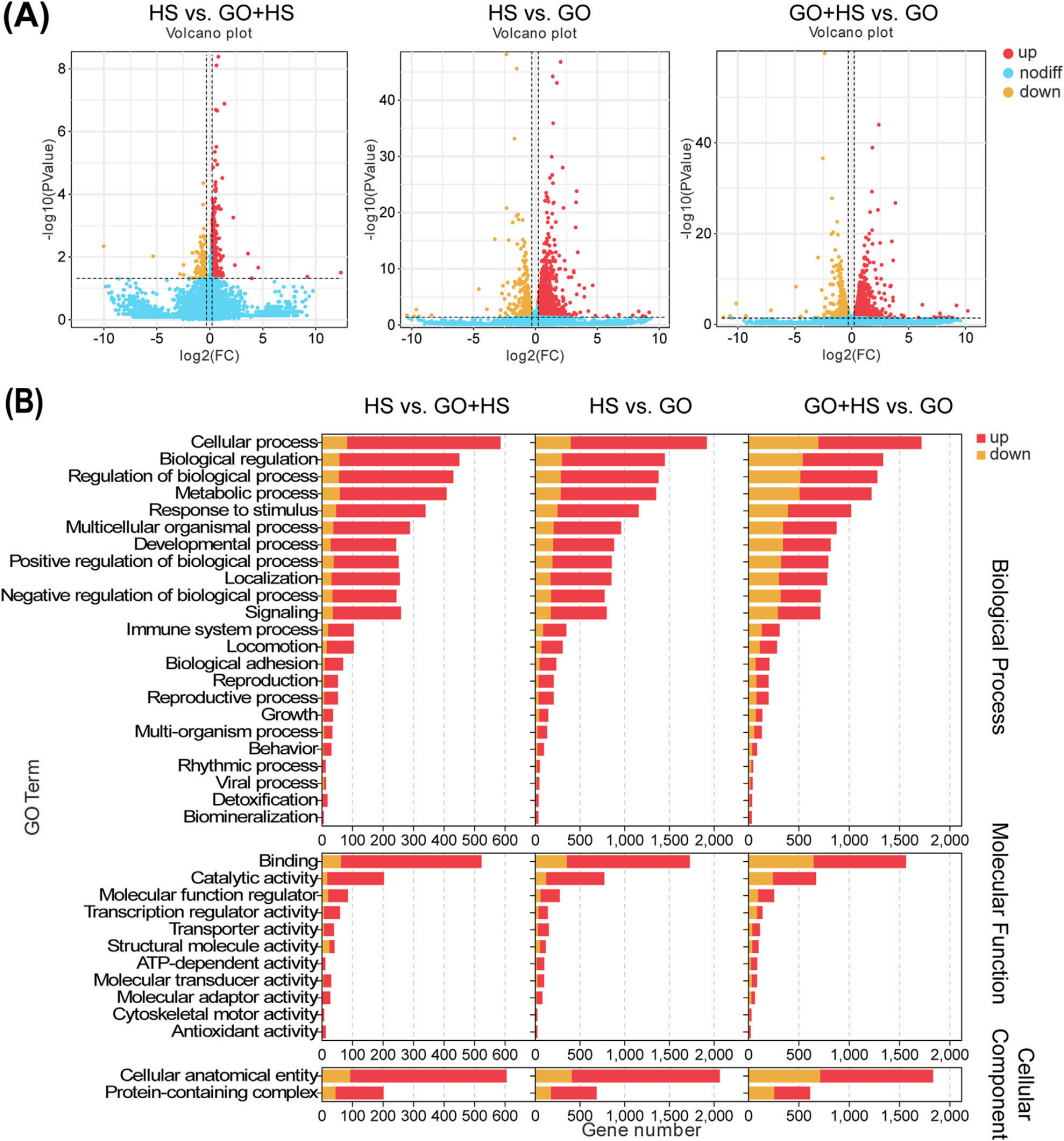
Summary of differentially expressed genes (DEGs) of C2C12 cells after 5 days of differentiation in cultures with horse serum, graphene oxide (GO), and GO combined with horse serum. **(A)** Volcano plot displaying an overview of the DEGs. HS represents the control group with 2% horse serum and no GO; GO + HS indicates the experimental group with GO and 2% horse serum. GO refers to the experimental group with GO but without horse serum. **(B)** GO channel enrichment statistical histogram.

The C2C12 cells were differentiated under three conditions: GO, horse serum, and GO combined with horse serum. RNA-Seq analysis revealed distinct gene expression changes in all the groups compared with those in the control group (containing 2% horse serum). In the GO plus horse serum group, 563 genes were upregulated and 93 were downregulated, whereas, in the GO-only group, 1,758 genes were upregulated and 425 were downregulated (Figure 7A).

**FIGURE 8:**
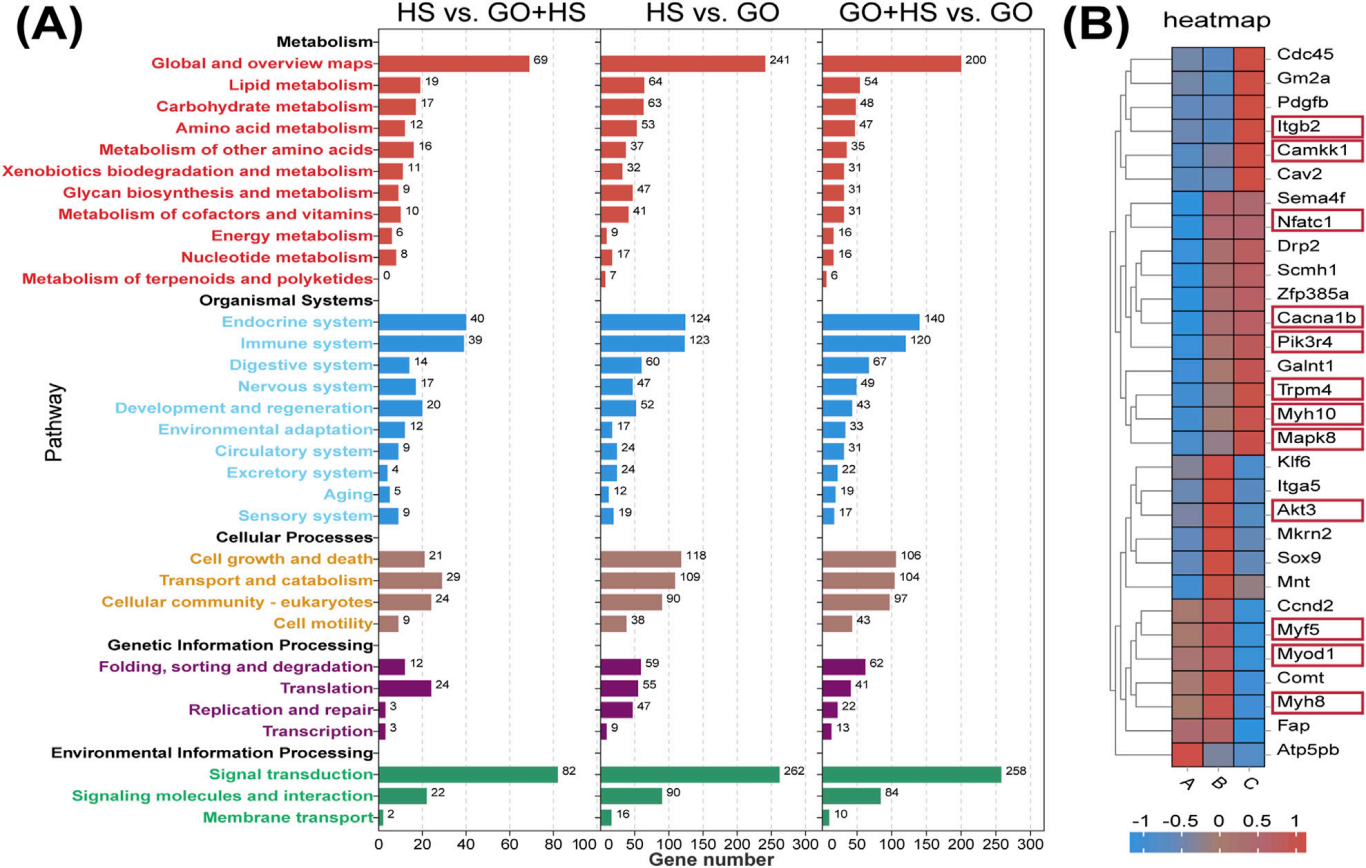
KEGG analysis results and differential gene expression analysis. **(A)** KEGG pathway enrichment histogram. **(B)** RNA-seq gene expression heat maps. **(A)** Control group with 2% horse serum and no graphene oxide (GO). **(B)** Experimental group with GO and 2% horse serum. **(C)** Experimental group with GO without horse serum.

GO treatment upregulated binding and catalytic activities, suggesting enhanced protein interactions and enzymatic activity. In cellular components, increase in the amount of cellular anatomical entities and protein-containing complexes indicates cytoskeletal remodeling. Biological processes remained dominated by cellular processes, biological regulation, and metabolism, implying enhanced differentiation and metabolic activity, especially in the GO plus horse serum group (Figure 7B). KEGG analysis revealed strong enrichment in metabolic and regulatory pathways, with horse serum partially mitigating GO-induced changes. GO significantly influenced signal transduction, with minor differences between the GO and GO plus horse serum groups, suggesting that horse serum modulates but does not override the effects of GO (Figure 8A).

In the GO plus horse serum group, the genes associated with muscle differentiation, including *MyoD1*, *Myf5*, and *MyH8*, were significantly upregulated, indicating enhanced myogenesis. Furthermore, the genes associated with the PI3K-Akt signaling pathway, such as *Pik3r4*, were upregulated in the GO plus horse serum and GO-only groups. *Akt3* was significantly upregulated, specifically in the GO plus horse serum group. The *Myo9b* gene, which is associated with cell adhesion, also exhibited elevated expression in both the treatment groups compared with that in the control group. The upregulation of *NFATc1* suggested that GO activated the NFAT signaling pathway, which has been implicated in the regulation of muscle growth and regeneration (Meissner et al., 2007). This activation is potentially linked to the enhanced electrical conductivity of GO, which can modulate intracellular signaling cascades. These findings collectively highlight the positive effects of GO and horse serum on myogenic differentiation and related signaling pathways (Figure 8B).

### 3.7 Effect of exosomes on MC3T3-E1 cells after C2C12 cell differentiation with or without GO

**FIGURE 9:**
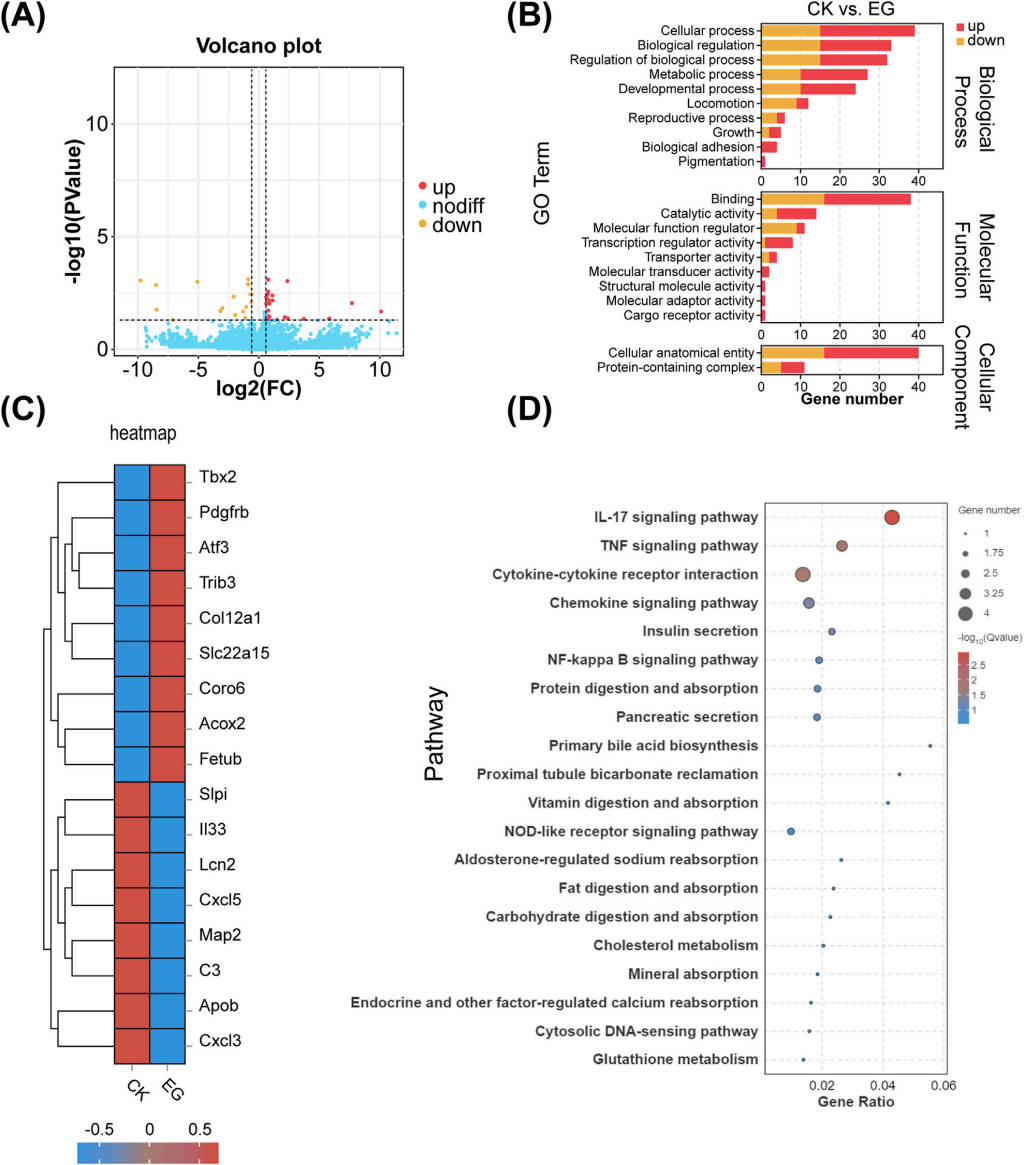
Effects of exosomes on MC3T3 cells after C2C12 cell differentiation with or without graphene oxide (GO). **(A)** Volcano plots. **(B)** Comparison of GO enrichment. **(C)** RNA-seq gene expression heat maps. **(D)** KEGG pathway concentration statistical bubble chart (CK represents the control group of MC3T3-E1 cells treated with normal medium, whereas, EG represents the experimental group of MC3T3-E1 cells treated with exosomes derived from C2C12 cells cultured with GO).

RNA-Seq analysis of MC3T3-E1 cells treated with the exosomes derived from C2C12 cells cultured with GO revealed the upregulation of 26 genes and the downregulation of 18 genes compared to that in the control group (Figure 9A).

The GO-treated exosomes exhibited changes in cellular processes, biological regulation, biological process regulation, and metabolic processes. This finding suggests that the exosomes derived from GO-cultured C2C12 cells affects the metabolic activities and growth of MC3T3-E1 cells. Additionally, changes in functions such as catalytic activity and other functional aspects were observed (Figure 9B). In the experimental group, significant upregulation was observed for genes such as *Tbx2* (involved in cell differentiation and development), *PDGFRB* (a receptor critical for cell proliferation), and *COL12A1* (a collagen essential for the ECM structure) (Chen et al., 2001; Bretaud et al., 2019; Khayal et al., 2018). Furthermore, *TRIB3* (involved in stress response and metabolic regulation), *SLC22A15* (a transporter), and *CORO6* (important for cell migration) were upregulated (Bai et al., 2021). Alternatively, genes such as *CXCL5* and *CXCL3* (encoding chemokines related to immune cell recruitment), *C3* (encoding a complement system component), and *ApoB* (encoding a lipoprotein negatively correlated with bone mineral density) were downregulated (Figure 9C) (Zhu et al., 2022). The differential genes are primarily enriched in the IL-17 and TNF signaling pathways (Figure 9D).

## 4 Discussion

Herein, GO promoted C2C12 myoblast proliferation at diameters of >500 nm and concentrations of 2.5 μg/mL. CCK-8 assay and flow cytometry analysis revealed that the cytotoxicity of GO is dose-dependent, with low concentrations exhibiting high biocompatibility (Mittal et al., 2016).

At the same concentration, GO with a larger diameter exhibited lower electrical conductivity (Figure 1C), which corresponded with the observed calcium ion influx across various concentration gradients (Figure 5). Notably, our evaluation of myogenic differentiation via PCR revealed that the expression of myogenic differentiation markers generally increased with increasing conductivity (across different sizes and concentrations of GO; Figures 6A,B). However, at a concentration of 2.5 μg/mL, the larger-sized GO (>500 nm) significantly enhanced myogenic gene expression than the smaller-sized GO. We speculate that this difference can be attributed to the superior biocompatibility of the larger GO sheets. GO exhibits excellent myogenic differentiation ability, mainly because of its electrical conductivity and surface morphology. Calcium ions (Ca^2+^) can activate *NFATc1* (Kar et al., 2011). Increased GO conductivity plays a significant role in supporting the expression of *MyoD* and enhancing myogenic differentiation, with effects likely mediated through the regulation of Ca^2+^ channels and the subsequent activation of intracellular signaling cascades. The conductivity of GO appears to influence membrane electrical activity, facilitating the activation of voltage-gated calcium channels on the surface of C2C12 cells. This process enables the influx of Ca^2+^ ions into cells, which is essential for the various signaling pathways involved in muscle differentiation. An increase in intracellular calcium concentration can stimulate the calcineurin/NFATc1 pathway. Calcineurin, a calcium/calmodulin-dependent phosphatase, is activated by an increase in Ca^2+^ ions. Upon activation, calcineurin dephosphorylates NFATc1, causing it to translocate into the nucleus where it acts as a transcription factor to regulate genes involved in muscle differentiation and growth. The upregulation of NFATc1 in response to GO treatment indicates that GO’s conductivity helps trigger this pathway, which is essential for the regulation of genes such as *MyoD*—an early marker and driver of myogenic differentiation (Zammit, 2017). Moreover, the conductive properties of GO enhance cellular membrane activity, likely promoting the sustained activation of PI3K-Akt (Xu et al., 2024). This pathway plays a pivotal role in regulating growth and differentiation by phosphorylating and activating various downstream targets, including myogenic regulatory factors, such as MyoD (Xu and Wu, 2000). These observations are consistent with those reported in previous studies highlighting the effects of conductive materials on skeletal muscle cell differentiation, wherein conductivity has been shown to improve gene expression and cell arrangement required for effective differentiation (Dong et al., 2020; Wang et al., 2021; Dong et al., 2017).

GO promoted the upregulation of adhesion-related genes, such as *ITGB2* and *GM2A*, in C2C12 cells. Cytoskeletal immunofluorescence results further confirmed that GO enhanced cell adhesion, likely due to the increased surface roughness of GO, which improves cell adhesion by providing more contact points and increasing the surface area for cell–material interactions (Taylor et al., 2017). This promotes stronger attachment, further promoting the diffusion of cells and the stability of the differentiation process.

**FIGURE 10:**
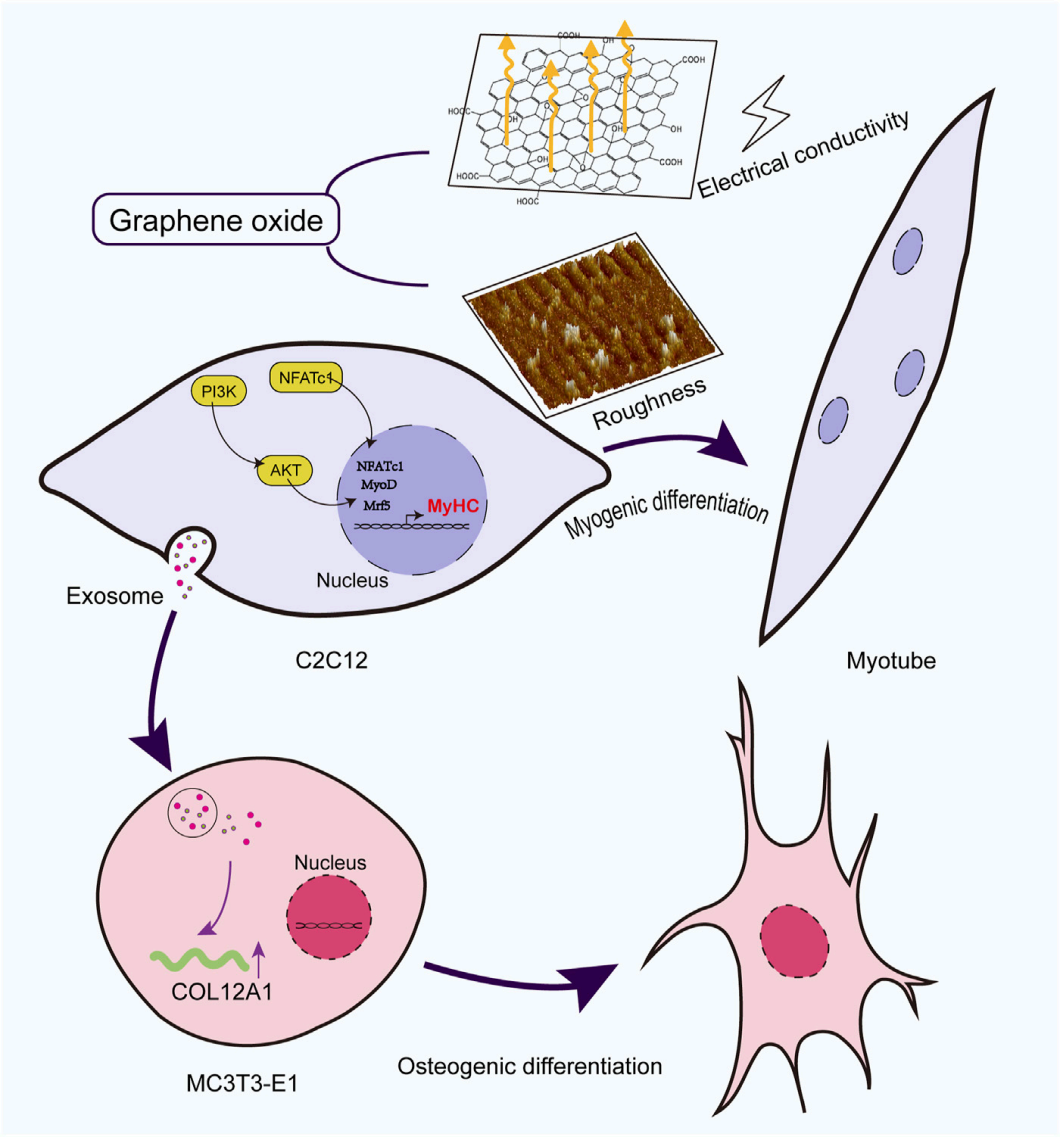
Mechanism diagram of GO promoting skeletal muscle complex regeneration.

Furthermore, previous studies have reported that GO-based materials promote osteogenic differentiation and antimicrobial activity in MC3T3-E1 osteoblasts (Daulbayev et al., 2022; Park et al., 2016; Chen et al., 2016). Despite these insights, limited research has been conducted on the effects of GO in the broader musculoskeletal system. Herein, MC3T3-E1 cells were cultured with the exosomes derived from C2C12 myoblasts differentiated in the presence of GO; these exosomes upregulated genes such as *PDGFRB*, *COL12A1*, and *TBX2*, while downregulating inflammation-related genes such as *C3* (Liu et al., 2022). This indicates that GO can help create a favorable microenvironment for bone formation and repair. The dual effects of GO on muscle and bone cells highlight its potential as a therapeutic strategy for addressing musculoskeletal complex injuries, degenerative diseases, and age-related declines in musculoskeletal integrity (Figure 10).

Future studies on GO applications should investigate its systemic effects on different cell types within the musculoskeletal system and potential immune responses to GO. Furthermore, exploring potential synergies between GO and other regenerative approaches, such as stem cell therapies and growth factor delivery, could further enhance muscle and bone tissue engineering outcomes. Another key area for further investigation is the role of the exosomes derived from C2C12 myoblasts in mediating GO’s beneficial effects on MC3T3-E1 cells. Analyzing the exosome cargo—including specific proteins, microRNAs, and metabolites—may reveal new signaling pathways and mechanisms that drive osteogenic differentiation and ECM formation. Identification of the factors associated with the exosomes responsible for these effects could lead to new therapeutic possibilities. Finally, previous studies have highlighted the significant potential of GO-based scaffolds for *in vivo* applications, particularly in the fields of tissue engineering and regenerative medicine (Jo et al., 2020; Gwon et al., 2024; Valencia et al., 2018). Based on these insights and our current findings, we believe that the GO formulation presented herein—with a diameter of >500 nm and a concentration of 2.5 μg/mL, which demonstrated the highest bioactivity *in vitro*—can be combined with other materials to construct composite scaffolds for the *in vivo* repair of skeletal muscle defects. However, the practical feasibility and therapeutic efficacy of this approach remain to be further validated through comprehensive animal studies.

## 5 Conclusion

GO with diameters of >500 nm and at concentrations of 2.5 μg/mL promotes balanced enhancement of C2C12 cell proliferation and myogenic differentiation. The increased electrical conductivity of GO stimulates calcium ion flow across the cell membrane and the upregulation of *NFATc1* and *MyoD* genes, thereby promoting the myoblast differentiation of C2C12 cells. GO enhances cell adhesion and promotes the upregulation of adhesion-related genes. Furthermore, the exosomes derived from GO-treated myoblasts upregulate genes such as *PDGFRB*, *COL12A1*, and *TBX2* while downregulating inflammation-related genes such as *C3*. These findings provide valuable insights into optimizing GO size, concentration, electrical conductivity, and surface roughness for improved biocompatibility and differentiation of both myoblasts and osteoblasts. This study highlights the potential of GO as a therapeutic material for simultaneous muscle and bone regeneration, facilitating the repair of the musculoskeletal complex.

## Data Availability

The original contributions presented in the study are included in the article/Supplementary Material, further inquiries can be directed to the corresponding authors.

## Abbreviations

AFM, Atomic force microscopy; BSA, Bovine serum albumin; DEG, Differentially expressed genes; DMEM, Dulbecco’s Modified Eagle Medium; ECM, Extracellular matrix; GO, Graphene oxide; PBS, Phosphate-buffered saline; HBSS, Hank’s Balanced Salt Solution; PCR, Polymerase chain reaction; PI, Propidium iodide; SEM, Scanning electron microscopy.
