# Supplementary material for: Evaluating the potential of graphene oxide to promote skeletal muscle complex regeneration
Source: Front Bioeng Biotechnol. 2025 Jul 31;13:1574145. doi: 10.3389/fbioe.2025.1574145 (PMC12350265; doi:10.3389/fbioe.2025.1574145)
Supplement: Supplementary file 1 [file Supplementaryfile1.docx]

Supplementary Material


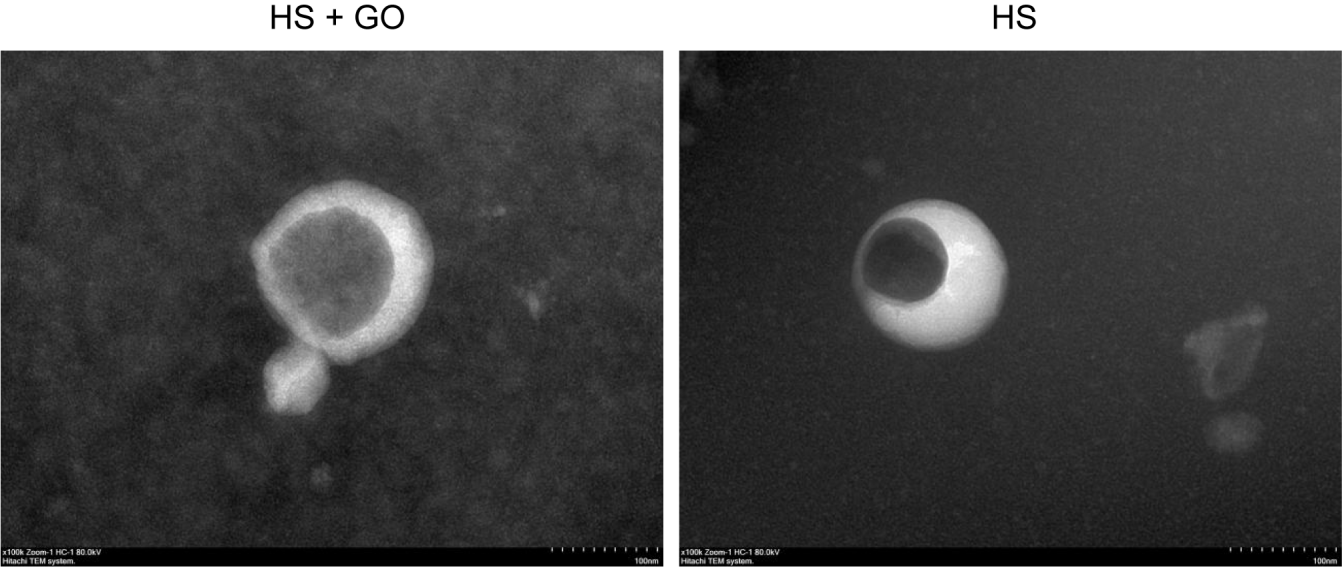


**Supplementary Figure 1.** TEM image of exosomes derived from C2C12 cells cultured with GO (HS: horse serum; GO: Graphene oxide)


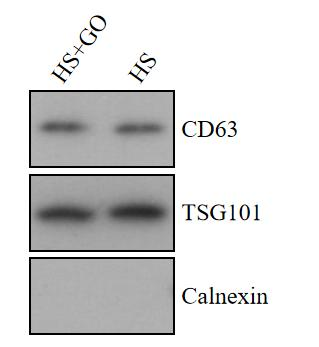


**Supplementary Figure 2.** Western blot results of exosomes derived from C2C12 cells cultured with GO


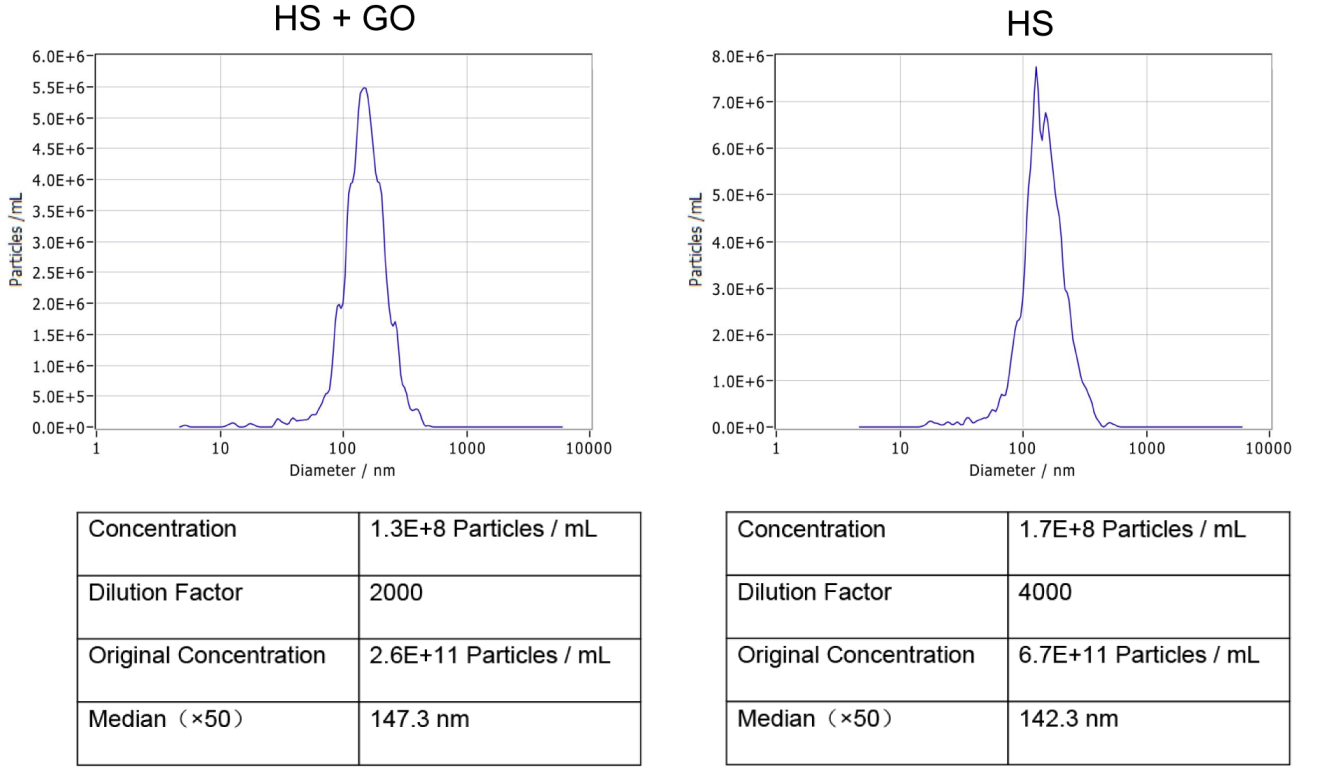


**Supplementary Figure 3.** Results of NTA data on exosomes derived from graphene oxide differentiation and culture of C2C12 cells

Exosomes extracted from C2C12 cells were successfully isolated and characterized in both the experimental group (cultured with GO) and the control group (cultured with 2% horse serum only). TEM results showed that exosomes from both groups had a cup-shaped morphology, but the exosomes in the experimental group were slightly larger in diameter compared to the control group (Supplementary Figure 1). Western blotting analysis detected the expression of CD63 and TSG101 in the exosome samples, while Calnexin expression was not detected (Supplementary Figure 2). CD63 and TSG101 are well-known exosome markers, while Calnexin is a protein associated with intracellular organelles. This result supports the purity of the exosome preparation, indicating that the isolation process effectively enriched exosomes and minimized contamination from other cellular components.

Nanoparticle tracking analysis (NTA) was used to evaluate the concentration, size distribution, and particle count of the isolated exosomes. For the control group (cultured with 2% horse serum), the particle concentration at a dilution factor of 4000 was 1.7E+8 particles/mL, with a raw concentration of 6.7E+11 particles/mL. The median particle size (×50) was 142.3 nm, within the typical exosome size range. In the experimental group (C2C12 cells cultured with GO), the particle concentration was slightly lower at 1.3E+8 particles/mL, with a dilution factor of 2000 and a raw concentration of 2.6E+11 particles/mL. The median particle size (×50) in the experimental group was 147.3 nm (Supplementary Figure 3). This suggests a slight difference in particle size distribution between the control and experimental groups, with the experimental group having a slightly larger median size.
